# Cognitive enhancing supplements and medications in United States Resident Physicians

**DOI:** 10.1186/s12909-022-03778-w

**Published:** 2022-10-27

**Authors:** Tyler Etheridge, Brandon Kennedy, Morgan M. Millar, Ben J. Brintz, Chaorong Wu, Jeff Pettey

**Affiliations:** 1 Department of Ophthalmology, John A. Moran Eye Center, University of Utah, 65 Mario Capecchi Dr, 84123 Salt Lake City, UT USA; 2 Division of Epidemiology, Department of Internal Medicine, University of Utah, Salt Lake City, UT USA

**Keywords:** Cognitive enhancement, Nootropics, Physicians, Residents, Supplements, Medications

## Abstract

**Background:**

The use of cognitive-enhancing medications and supplements among healthy adults continues to rise. Limited data exists on their use among resident physicians. Given their highly competitive and stressful lifestyle, we sought to evaluate the prevalence, motivations, and side effects of using cognitive-enhancing supplements and medications among resident physicians at a large United States academic institution.

**Methods:**

An anonymous web-based survey was circulated to resident physicians inquiring about using cognitive-enhancing supplements and medications, as well as personal characteristics such as gender, marital and parental status, medical diagnoses, and medical specialty. Before circulation, we performed a pilot study. Weighted logistic regression analyses estimated the impact of personal characteristics on the probability of using both supplements and medications.

**Results:**

Survey response rate was 46.4%. Of respondents, 48.6% were female, 45.9% were married, 70.9% were without children, and 67.2% were in a non-surgical medical specialty. Few respondents had a related medical diagnosis, with attention deficit hyperactivity disorder being the most common (7.1%). Male, non-married, surgical residents were more likely to take supplements (odds ratio (OR) = 1.06, 1.05, and 1.05). Males, without children, and those who felt pressure to perform well, were afraid of being left behind, felt pressure because colleagues take them, or felt they could not reach their current level of training without medications were more likely to take medications (OR = 1.11, 1.04, 1.05, and 1.08). Adverse effects with medications were common.

**Conclusion:**

Supplement and medication use for cognitive enhancement was high among resident physicians at a single institution despite few having a related medical diagnosis. This study raises awareness of the growing pressure in competitive residency environments to use cognitive enhancement regardless of the potential side effects.

**Supplementary Information:**

The online version contains supplementary material available at 10.1186/s12909-022-03778-w.

## Background

Prescription medications to enhance cognitive performance among healthy adults continues to gain popularity [1–3]. Cognitive enhancers are taken to improve memory, boost levels of energy and wakefulness, and increase mental alertness and concentration [4]. The mechanisms of action of these medications are often inconclusive, with some working to increase levels of circulating adrenalin to assist with wakefulness or modulate neurotransmitters such as dopamine. The most common medications include stimulants traditionally prescribed for attention deficit hyperactivity disorder (ADHD). Over 6% of healthy individuals use stimulants [5], with even higher rates among college students [6–9], medical students [10–14], and resident physicians [15]. Although the motivations may vary, common ones include a stressful lifestyle, a competitive environment, balancing the rigors of academic or professional obligations and social expectations, and a fear of being “left behind” by peers [15–17].

In addition to prescription medications, there is a growing list of over-the-counter supplements marketed as cognitive enhancers [4]. Collectively, these have been coined under the umbrella term, nootropics, which have gained popularity as international sales continue to climb and are expected to reach USD$5.32 billion by 2026 [18]. However, the use of medications and supplements for cognitive enhancement among resident physicians in the United States has been underexplored. Resident physicians are a unique population with a broad range of stressors that may influence their likelihood of using cognitive-enhancing medications and supplements. Stressors include long work hours, chronic sleep deprivation and fatigue, personal debt incurred from increasing medical education costs, decreasing confidence in the job market, and increasing efforts to master a rapidly expanding knowledge base in the more litigious medical landscape [19]. Furthermore, substance misuse, particularly illicit substance abuse has been associate with increased rates of suicide, an issue of significant concern among medical trainees and practicing physicians [20] . Therefore, we evaluated the prevalence, motivations, and side effects of cognitive-enhancing supplements and medications among resident physicians at a large United States academic institution.

## Methods

We distributed an anonymous, cross-sectional, voluntary survey on the use of cognitive-enhancing supplements and medications to all 638 resident physicians at the University of Utah, a large public United States academic institution with over 23,700 employees and over 33,000 students. The institution is in the urban setting of Salt Lake City, Utah. A resident is a physician who has completed their medical degree and is undergoing additional training in a medical specialty of choice as part of a graduate medical education program. We distributed the survey using a web-based platform in January of 2022 via email on three separate occasions to all resident physicians at the University of Utah. There was no survey incentive provided. All respondents expressed written consent by completing the survey and were asked to complete the survey only once. The survey design included a pilot study of randomly selected residents from multiple specialties representing 5% of the study population (n = 30). Pilot study respondents completed the survey within approximately 5 minutes. The response rate of the pilot study was 43.3% (13 of 30 residents). Pilot survey respondents provided anonymous feedback about the survey. We incorporated all suggestions in revisions to the final survey.

Survey sections included demographic information; cognitive enhancing supplement information, including type, duration, frequency of use, side effects, and motivations; cognitive-enhancing medication information, including type, duration, frequency of use, side effects, and motivations. We limited the collection of demographic information to those variables previously shown to be associated with cognitive-enhancing medication use among resident physicians to ensure the anonymity of respondents [15]. Respondents were asked at what stage of their medical training did they start taking specific medications, including Amphetamine, Methylphenidate, and Modafinil, as well as particular supplements, including Noopept and Racetams. These medications and supplements were asked about because of their explicit use as cognitive enhancement. In addition, a follow-up question about the current frequency of use was utilized to determine whether respondents were currently using the medication or supplement or had simply used it in the past (Supplement).

Specific cognitive enhancing supplements measured included Noopept, Racetams (piracetam, pramiracetam, phenylpiracetam or phenotropil, aniracetam), Ashwagandha, Bacopa monnieri, Caffeine, Creatine, Ginkgo biloba, Lion’s Mane Mushroom, L-theanine, magnesium, Omega-3 fatty acids, Panax ginseng, and Rhodiola Rosea. Although many of the supplements, such as Omega-3 fatty acids, can be used for other purposes, respondents were asked explicitly about their use for cognitive enhancement. In addition, the form of caffeine consumption was not inquired about. Cognitive enhancing medications included Amphetamines (such as Adderall, Adzenys, Desoxyn, Dexedrine, Dyanavel, Evekeo, Mydayis, ProCentra, Vyvanse, or Zenzedi), Methylphenidates (such as Adhansia, Azstarys, Aptensio, Concerta, Contempla, Daytrana, Focalin, Journal, Metadate, Methylin, QuilliChew, Quillivant, or Ritalin), Modafinil (Provigil), Cholinesterase inhibitors [such as Donepezil (Aricept), Rivastigmine (Exelon), or Galantamine (Razadyne)], Glutamate regulator [Memantine (Namenda)], and Cholinesterase inhibitor + glutamate regulator [Donepezil and memantine (Namzaric)]. The survey also allowed participants to add ‘other’ cognitive enhancing supplements and medications.

We asked survey respondents about related medical diagnoses, including learning disorder or disability, ADHD, shift work sleep disorder, narcolepsy, sleep apnea with excessive daytime sleepiness, and neurodegenerative diseases such as Alzheimer’s disease or dementia. We also asked respondents about their motivations and perceptions of cognitive-enhancing supplements or medications. The University of Utah Institutional Review Board (IRB) approved the survey (Supplement). The study adhered to the Declaration of Helsinki.

We reported descriptive statistics of respondent demographics and stimulant use using counts and percentages out of the total for each question and out of the subset for nested questions. To evaluate the prevalence of using cognitive enhancing supplements or medications and the reasons, we used the raking procedure to weight our completed sample to represent the population of interest [21]. We only used gender for calibration due to the desire to keep the survey respondents anonymous. We present estimates and confidence intervals for the estimated prevalence of the population that uses the supplements and medications.

We used weighted multivariable logistic regression to estimate the impact of personal characteristics on the probability of using supplements and medications for improving cognitive performance. These characteristics included gender, marital status, parental status, and motivations and perceptions of use. Response variables included the use of supplements (either Noopept or Racetams) and the use of medications (Amphetamine, Methylphenidate, or Modafinil). We reported odds ratios (OR) and confidence intervals. A two-tailed alpha level of 0.05 was selected. We performed data analysis with R-4.1.3, using the R package ‘Survey’ for the raking procedure and the weighted logistic regression.

## Results

According to institutional records of the resident demographics at the time of our study, our survey population consisted of 638 resident physicians with an average age of 31 years. The population consisted of 315 males (49.4%), 317 females (49.7%), and 6 unknown (0.9%). The most common ethnicity was White or Caucasian at 477 (74.8%), followed by Asian at 61 (9.6%), two or more races at 35 (5.5%), Hispanic/Latino/Spanish at 26 (4.1%), and unspecified at 26 (4.1%).

Of the 638 asked to participate, 296 (46.4%) residents completed the survey. Table 1 shows unweighted descriptive statistics summarizing the participant demographics. Among respondents, there were 144 females (48.6%), 120 males (40.5%), and 32 unknown (10.8%). One hundred and twenty-nine (45.9%) residents were married, and 54 (18.2%) had children. Most respondents (67.2%) were in a non-surgical medical specialty.

**Table 1:** Participant demographics

Variable		Sample N (%) (Total = 296)
Gender	Female	144 (48.6%)
	Male	120 (40.5%)
	Unknown	32 (10.8%)
Married	Yes	136 (45.9%)
	No	129 (43.6%)
	Prefer not to answer	1 (0.3%)
Children	Yes	54 (18.2%)
	No	210 (70.9%)
	Prefer not to answer	2 (0.7%)
Primary Specialty	Anatomic Pathology and Clinical Pathology	14 (4.7%)
	Anesthesiology	20 (6.8%)
	Dermatology	2 (0.7%)
	Diagnostic Radiology	8 (2.7%)
	Emergency Medicine	9 (3.0%)
	Family Medicine	8 (2.7%)
	General Surgery	13 (4.4%)
	Internal Medicine	49 (16.6%)
	Internal Medicine-Pediatrics	4 (1.4%)
	Internal Medicine	1 (0.3%)
	Neurological Surgery	5 (1.7%)
	Neurology	7 (2.4%)
	Obstetrics and Gynecology	11 (3.7%)
	Ophthalmology	12 (4.1%)
	Orthopedic Surgery	11 (3.7%)
	Otolaryngology	4 (1.4%)
	Pediatrics	34 (11.5%)
	Physical Medicine and Rehabilitation	7 (2.4%)
	Plastic Surgery	7 (2.4%)
	Prefer not to answer	14 (4.7%)
	Psychiatry	17 (5.7%)
	Radiation Oncology	1 (0.3%)
	Triple Board (Pediatrics, Psychiatry, Child and Adolescent Psychiatry)	4 (1.4%)
	Urology	4 (1.4%)
Specialty	Surgical	67 (22.6%)
	Non-surgical	199 (67.2%)
Learning disorder or disability	No	265 (89.5%)
	/	31 (10.5%)
Attention deficit hyperactivity disorder (ADHD)	Yes	21 (7.1%)
	No	245 (82.8%)
	/	30 (10.1%)
Shift work sleep disorder	Yes	23 (7.8%)
	No	242 (81.8%)
	/	31 (10.5%)
Narcolepsy	Yes	2 (0.7%)
	No	263 (88.9%)
	/	31 (10.5%)
Sleep apnea with excessive daytime sleepiness	Yes	4 (1.4%)
	No	261 (88.2%)
	/	31 (10.5%)
Neurodegenerative disease	No	265 (89.5%)
	/	31 (10.5%)

Note: ‘/’ is missing value. Data represent unweighted percentages

The most common medical specialty was Internal Medicine (16.6%), followed by Pediatrics (11.5%), and Anesthesiology (6.8%). Twenty-one (7.1%) respondents reported a history of ADHD, 23 (7.8%) shift work sleep disorder, 2 (0.7%) narcolepsy, and 4 (1.4%) sleep apnea with excessive daytime sleepiness.

**Fig. 1:**
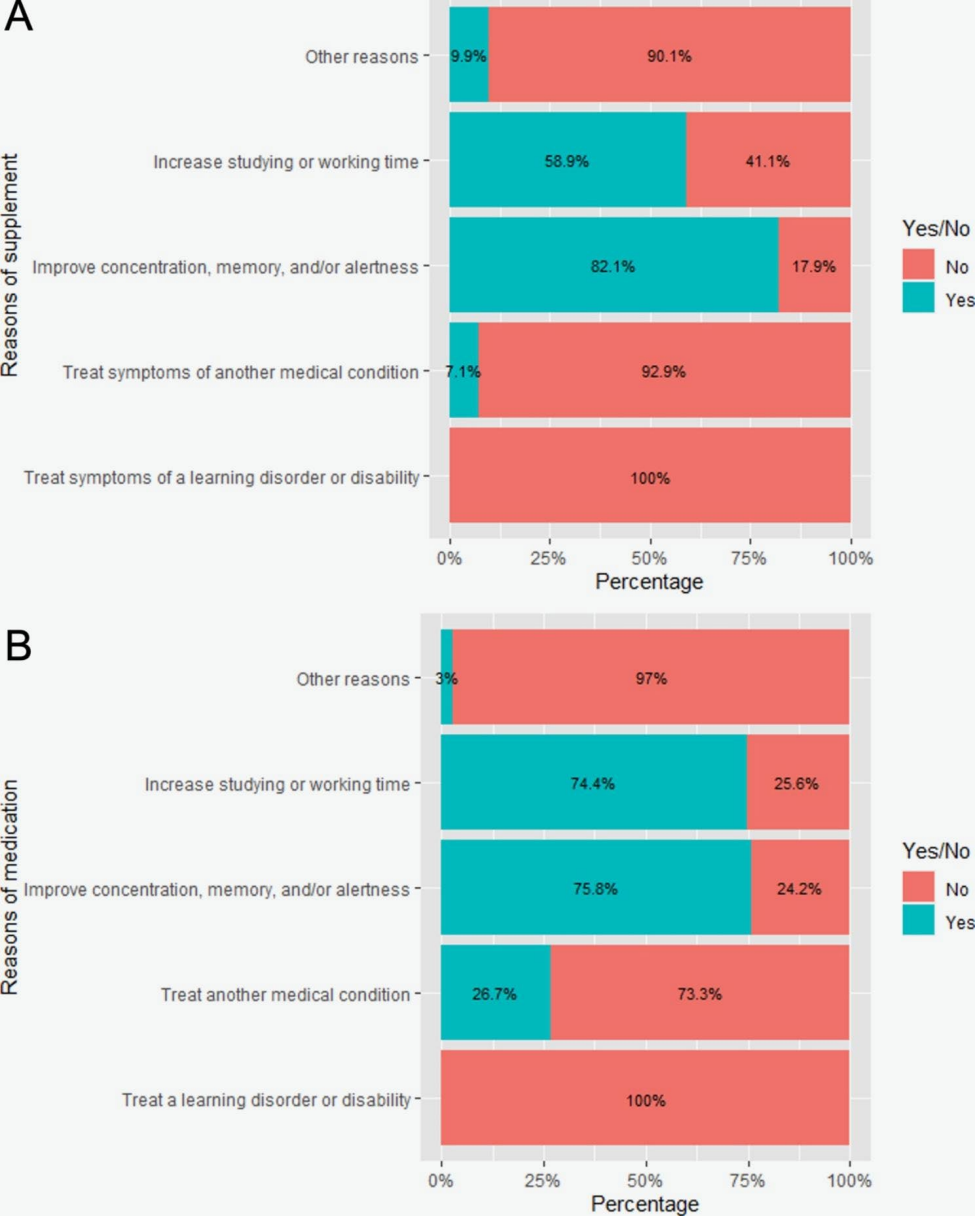
Reasons for cognitive enhancing supplement (**A**) and medication (**B**) use (for those who used)

Using weighted analysis, we estimate the prevalence of caffeine use at 78.4%, the most among all the supplements (Table 2). Omega-3 fatty acids (9.9%), creatine (8.9%), and Lion’s Mane Mushroom (5.3%) were also common. Amphetamine and Modafinil were used the most among cognitive-enhancing medications at 19.2% and 11.1%, respectively. No residents used Glutamate regulators or Cholinesterase inhibitor + Glutamate regulators. To improve concentration, memory, or alertness and to increase studying or working time were the most common reasons for taking cognitive-enhancing supplements (82.1% and 58.9%) and medications (75.8% and 74.4%) (Fig. 1). Unfortunately, we observed a high non-response rate for questions asking when residents began taking supplements (97.3%) and medications (99.0%) and how frequently they use supplements (82.8%) and medications (82.8%) for cognitive enhancement.

**Table 2:** Weighted prevalence of cognitive enhancing supplement and medication use

	Prevalence (N = 296)	
**Supplements**	**%**	**95% CI**
Noopept	0.8%	(0.3%, 1.9%)
Racetams	3.3%	(1.9%, 5.1%)
Ashwagandah	3.8%	(2.3%, 5.7%)
Bacopa monnieri	0.5%	(0.1%, 1.3%)
Caffeine	78.4%	(74.7%, 81.9%)
Creatine	8.9%	(6.6%, 11.7%)
Ginkgo biloba	1.6%	(0.7%, 2.9%)
Lion’s Mane Mushroom	5.3%	(3.5%, 7.5%)
L-theanine	3.4%	(2.1%, 5.2%)
Magnesium	3.8%	(2.4%, 5.8%)
Omega-3 fatty acids	9.9%	(7.5%, 12.8%)
Panax ginseng	1.2%	(0.5%, 2.5%)
Rhodiola rosea	1.6%	(0.8%, 3.0%)
Other	4.7%	(3.0%, 6.8%)
**Medications**		
Amphetamine	19.2%	(15.9%, 22.9%)
Methylphenidate	6.3%	(4.4%, 8.7%)
Modafinil	11.1%	(8.5%, 14.0%)
Cholinesterase inhibitor	0.3%	(0.1%, 1.1%)
Glutamate regulator	0.00%	/
Cholinesterase inhibitor + glutamate regulator	0.00%	/

Note: ‘/’ is missing value

No residents reported side effects with supplement use, specifically with Noopept and Racetams. However, of those who reported taking cognitive-enhancing medications, 25 (49.0%) reported side effects with Amphetamine, including change in appetite (84.0%), sleeplessness (48.0%), euphoria or heightened sense of being (40.0%), anxiety or paranoia (36.0%), palpitations (28.0%), and headache (36.0%). Of the eight (50.0%) who reported taking Methylphenidate, sleeplessness (75.0%), change in appetite (37.5%), euphoria or heightened sense of being (37.5%), anxiety or paranoia (12.5%), and palpitations (12.5%) were the most common. Twelve (42.9%) residents reported adverse effects with Modafinil, including nausea or vomiting (66.7%), dizziness (16.7%), change in appetite (8.3%), euphoria or heightened sense of being (8.3%), palpitations (8.3%), and sleeplessness (8.3%).

**Fig. 2:**
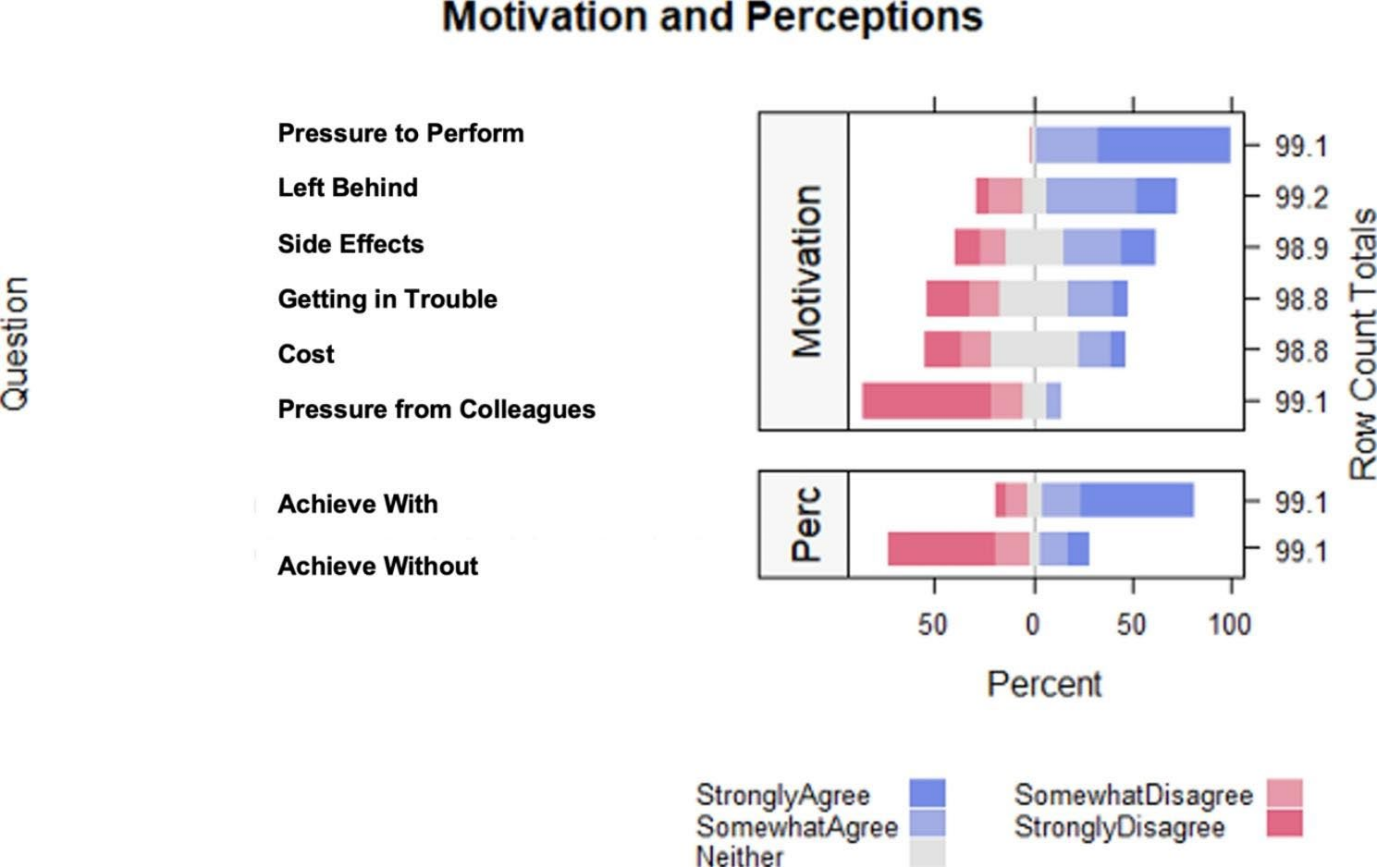
Motivations and perceptions for cognitive enhancing supplement and/or medication use

Figure 2 shows the distribution of motivations and perceptions with the statement asked on the y-axis and the percentage of respondents on the x-axis. The percentage of respondents who agree with the statement is represented to the right of the zero line, while the percentage of respondents who disagree is shown to the left. The percentage of respondents who neither agree nor disagree is split down the middle and represented in a neutral color. The categories within each sector are ordered by the percentages who agree. Residents largely agreed (strongly agree and somewhat agree) with “I feel pressure to perform well professionally or academically,“ “I feel afraid that I will be left behind professionally or academically,“ and “It is possible to achieve the level of academic or professional performance expected of me without taking a cognitive enhancing supplement(s) or medication(s).“ Most disagreed (strongly disagree and somewhat disagree) with “I feel pressure to take cognitive-enhancing supplements or medications because my colleagues take them” and “I could not have reached my current level of training without taking a cognitive-enhancing supplement(s) or medication(s).“

**Fig. 3:**
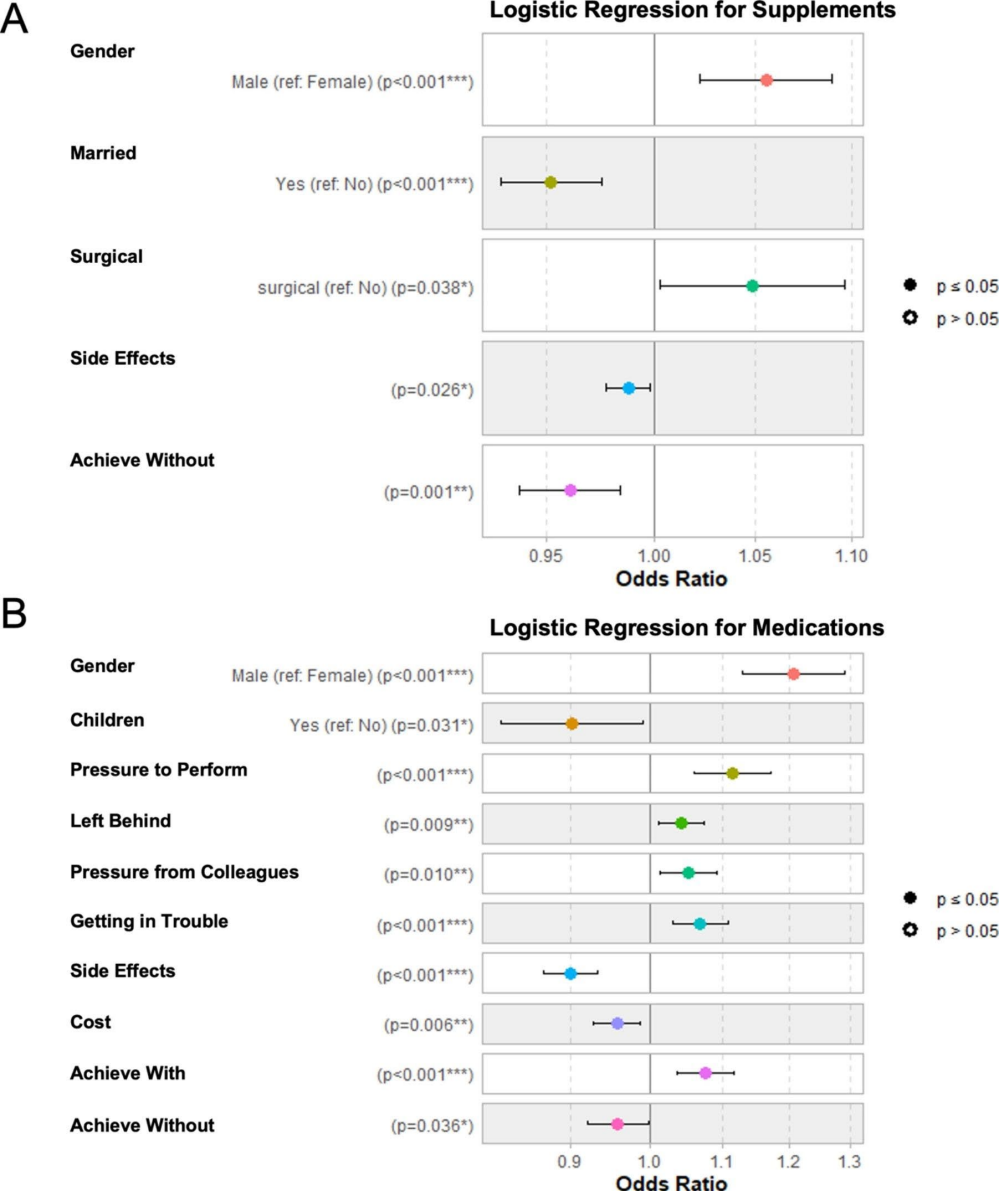
Odds ratios (only for significant predictors) for use of cognitive enhancing supplements (**A**) and medications (**B**)

Gender, marital status, medical specialty (surgical versus non-surgical), feeling nervous about the side effects of cognitive-enhancing supplements, and believing they could achieve the level of academic or professional performance without taking cognitive enhancing supplements had strong evidence of an association with using cognitive enhancing supplements (Fig. 3 A). Specifically, the odds for males were 1.06 times higher than females, p < 0.01. The odds for non-married residents were 1.05 times higher than married residents, p < 0.01. The odds for surgical residents were 1.05 times higher than non-surgical residents, p = 0.04. Residents who felt nervous about the side effects of cognitive-enhancing supplements or who believed they could achieve the level of academic or professional performance without taking them were less likely to take them, with OR of 0.99 and 0.96 (p-values of 0.03 and < 0.01), respectively.

Gender, parental status, and all six motivation and perception survey items had strong evidence of an association with using cognitive-enhancing medications (Fig. 3B). Specifically, the odds for males were 1.21 times higher than females, p < 0.01. The odds for those without children were 1.11 times higher than those with children, p = 0.03. Residents who felt pressure to perform well, felt afraid of being left behind, felt pressure because their colleagues take them, felt nervous about getting into trouble if taking medications, or felt they could not reach their current level of training without taking medications were more likely to take cognitive enhancing medications with OR of 1.11, 1.04, 1.05, 1.07, and 1.08 (p-values of < 0.01, 0.01, 0.01, < 0.01, and < 0.01), respectively. Residents who felt nervous about the side effects, felt hesitant about the cost, or who believed they could achieve the level of academic or professional performance without taking cognitive-enhancing medications were less likely to take them, with OR of 0.9, 0.96, and 0.96 (p-values of < 0.01, 0.01, and 0.04).

## Discussion

Cognitive-enhancing supplements and medications, also known as nootropics, to increase mental alertness, improve memory, and boost levels of energy and wakefulness in healthy individuals are on the rise [1–3]. With common motivations, including a stressful lifestyle, working in a competitive environment, and attempting to balance the rigors of academic or professional obligations and social expectations [15–17], the prevalence of cognitive-enhancing medication use among resident physicians is over 20% [15]. A prior study examining cognitive-enhancing medication use among resident physicians in Israel [15], focused on stimulants, specifically amphetamines and methylphenidate. However, other cognitive-enhancing medications, such as Modafinil and Glutamate regulators or Cholinesterase inhibitor + Glutamate regulators, have yet to be explored. In addition, our work represents the most comprehensive survey of cognitive enhancing supplement use among United States medical residents.

In our completed sample, supplements for cognitive enhancement were frequent with caffeine, omega-3 fatty acids, creatine, and Lion’s Mane Mushroom most reported. Interestingly, despite a robust list of possible supplements, residents added to these options with the most common ‘other’ supplement being nicotine. We surveyed multiple cognitive-enhancing medications and similar to other studies of resident physicians [15], prevalence of Amphetamine use was approximately 20% despite only 7% reporting a history of ADHD. Methylphenidate, another stimulant, was infrequently used, and unsurprisingly almost no respondents used cholinesterase inhibitors or glutamate regulators, commonly prescribed for Alzheimer’s disease and dementia. Newer medications, such as Modafinil which treats narcolepsy, sleep apnea, and shift work sleep disorder by promoting wakefulness and alertness was reported just over 11% use by residents. Unfortunately, over 95% of respondents did not indicate when they began using cognitive-enhancing supplements or medications and over 80% did not report how frequently they take them. It’s unclear why respondents did not complete these critical questions. It’s unlikely that respondents were confused by the questions given all pilot study respondents completed these questions. Potentially, the lack of response was due to fear of stigmata about onset and frequency of use.

Claims about the cognitive benefits of supplements and medications are poorly supported, and the health risks posed by their use may be significant. For example, a growing body of research supports the long-term alterations in brain plasticity and dependence on stimulants with frequent use [22]. In addition, the association of substance misuse and increased rates of suicide poses a serious concern as suicide continues to be a common cause of death for medical trainees, including residents [20]. Although no respondents reported side effects with supplements, we only focused on the side effects of Noopept and Racetams, which had a reasonably low prevalence. We chose to focus our queries on the side effects of these supplements because of their singular purpose of cognitive enhancement, as opposed to other supplements such as omega-3 fatty acids with other reported benefits. In addition, to maximize the survey response rate by minimizing survey duration, we were limited in the number and length of questions. However, a significant percentage of residents who reportedly used medications for cognitive enhancement experienced adverse effects. These ranged from a change in appetite, euphoria or heightened sense of being, anxiety or paranoia, headache, sleeplessness, nausea and vomiting, dizziness, and palpitations. Although it is unclear whether side effects directly influenced these medications, fear of medication side effects was associated with a lower likelihood of use.

Prior studies have examined several motivations for using cognitive-enhancing medications. In our completed sample, the two key motivations for using supplements and medications were to improve concentration, memory, or alertness and increase studying or working time. Residents who were male, not married, and were in surgical specialties were more likely to use supplements. Whereas residents who felt nervous about the side effects of supplements or who believed they could achieve the level of academic or professional performance without them were less likely to use them. Residents who were male or did not have children were more likely to take medications, as were those who felt pressure to perform well, felt afraid of being left behind, felt under the pressure of colleagues, or felt they could not have reached their current level training without taking the medication. Those who were nervous about the side effects, felt hesitant about the cost, and believed they could achieve the level of academic or professional performance without taking cognitive-enhancing medications were less likely to use them.

Male residents in our study were more likely to use cognitive-enhancing supplements and medications. Unfortunately, prior research on the use of cognitive-enhancing medications among resident physicians by Rubin-Kahana et al. does not shed light on the reason that males are more likely to use nootropics [15]; however, men are more likely to use illicit drugs and alcohol [23]. In addition, single male respondents without children were more likely to use cognitive-enhancing supplements and medications. Marriage and parenting seem to have a protective effect on using illicit drugs, alcohol, and tobacco, which may explain these data [24]. Given those critical motivations for using cognitive-enhancing supplements and medications were to improve concentration, memory, or alertness and to increase studying or working time; it makes sense that residents who were in surgical specialties, such as neurosurgery, were more likely to use cognitive enhancers when compared to non-surgical specialties, such as internal medicine. Surgical residencies tend to be among the most competitive specialties, according to the National Resident Matching Program (NRMP) results [25]. Surgical specialties also tend to have longer work hours and carry the additional demands of being on call, meaning residents are required to be available at any time in addition to regular working hours [26].

Comparing the incidence of cognitive-enhancing medication use in our population of resident physicians with a similarly demanding profession, law, demonstrates that rates of use are similar [27]. Specifically, between 9% and 18% of law students are reported to use cognitive-enhancing medications, most commonly Adderall and Ritalin, to improve academic performance. In addition to a similar incidence of use, motivations for use among law students were similar. Reasons listed by law students included improved concentration, increased studying and working time, increased mental alertness while studying, and preventing other students from having an academic edge over them.

Our study had several limitations. First, our survey was newly developed and could exhibit measurement errors. There is no existing, validated instrument to capture motivations for the use of cognitive-enhancing supplements and medications. However, we followed best practices in questionnaire development and performed a pilot study on approximately 5% of our target population. The feedback given by pilot study participants was incorporated into the final survey to improve accuracy. Second, this was a self-reported survey with the possibility of misreporting. However, the rates of misreporting sensitive behaviors in anonymous web-based surveys are reportedly low [28]. Our study population consisted of resident physicians at a single large urban United States academic institution, and results may not apply to other populations. In addition, our study did not inquire about ethnicity to protect respondents’ anonymity, therefore no sub-analysis was conducted due to this risk. Given the high degree of variability among residency program competitiveness, even at a single institution, it is difficult to draw conclusions about the competitive nature of the University of Utah and how it applies across residency programs throughout the United States. Although we surveyed resident physicians from multiple medical specialties, some specialties had a low number of respondents. Given that little is known about survey non-responders, we cannot rule out the presence of non-response bias. However, we did have a robust response rate of over 46%. Finally, although data suggests there may be cognitive benefits to stimulant and modafinil use [29, 30], we did not examine the benefits of cognitive-enhancing supplements and medications among resident physicians. Given modafinil has been shown to reduce cognitive errors in sleep-deprived residents [30], future research on the benefits and side effects of its use are needed. In addition to future research, the authors hope that residency programs can use the risk factors outlined in our study to create a screening protocol to identify residents at risk of abusing cognitive-enhancing medications and supplements. Screening protocols would allow programs to allocate additional resources to at risk residents, thereby minimize their use.

## Conclusion

In conclusion, there was a high rate of supplement and medication use for cognitive enhancement among resident physicians at a single United States academic institution despite very few carrying a related medical diagnosis. Side effects of medications were not uncommon, and some had potentially serious health implications. Male residents in surgical specialties who were not married and did not have children were more likely to use cognitive enhancers. Those who used were more likely to feel pressure to perform well, feel afraid to be left behind, feel pressure from colleagues, or feel that they could not have reached their current level of training without them. Given the potential short- and long-term impact of their use, we need more research on the effects of cognitive-enhancing supplements and medications. This study raises awareness of the increasing pressure individuals feel in competitive residency environments to turn to cognitive enhancement, despite few having a related medical diagnosis. The motivations to use cognitive enhancers, regardless of the significant risk of potential adverse effects, legal ramifications, fairness, and ethics of use, must be addressed. Therefore, in an era of increasing burnout, suicide, and substance abuse, we recommend institutions use the risk factors identified in our study to create and implement screening protocols to identify residents at risk for cognitive-enhancing medication and supplement misuse and potential harm.

## Electronic supplementary material

Below is the link to the electronic supplementary material.

Supplementary Material 1

## Data Availability

The dataset used and/or analyzed during the current study are available from the corresponding author on reasonable request.

## List of abbreviations

ADHD: Attention deficit hyperactivity disorder.
IRB: Institutional review board.
NRMP: National Resident Matching Program.
OR: Odds ratios.
