# Supplementary material for: Cognitive enhancing supplements and medications in United States Resident Physicians
Source: BMC Med Educ. 2022 Oct 27;22:744. doi: 10.1186/s12909-022-03778-w (PMC9615194; doi:10.1186/s12909-022-03778-w)
Supplement: Supplementary file 1 — Supplementary Material 1 [file 12909_2022_3778_MOESM1_ESM.docx]

**Consent**

1. I’m going to ask you questions about cognitive supplements and medications.

This is an anonymous and voluntary survey. The information you provide will not be linked to your identity in any way including in your medical, professional, or personal record. Any questions about the survey may be emailed to Tyler Etheridge at [tyler.etheridge@hsc.utah.edu](mailto:tyler.etheridge@hsc.utah.edu). Thank you for your participation!

Are you willing to take this survey?

1. Yes
2. No

**Supplements**

1. Do you currently take, or have you ever taken any of the following supplements for the purpose of improving your cognitive performance? Please select yes or no for each.
2. Noopept
3. Racetams (piracetam, pramiracetam, phenylpiracetam or phenotropil, aniracetam)
4. Ashwagandah
5. Bacopa monnieri
6. Caffeine
7. Creatine
8. Ginkgo biloba
9. Lion’s Mane Mushroom
10. L-theanine
11. Magnesium
12. Omega-3 fatty acids
13. Panax ginseng
14. Rhodiola rosea
15. Other (please specify)

**Question 3-6 will be asked for Noopept and Racetams selected above.**

1. At what stage of your training did you start taking [pipe in name of specific supplement]?
2. Elementary school
3. Middle school
4. High school
5. College
6. Graduate school
7. Medical school
8. Residency
9. Other (please specify)
10. How frequently are you currently taking [pipe in name of specific supplement]?
11. No longer taking
12. Daily
13. At least once per week
14. At least once per month
15. At least once per year
16. Other (please specify)
17. Have you ever experienced side effects from taking [pipe in name of specific supplement]?
18. Yes
19. No
20. [if yes to 5] What side effects have you experienced? Select all that apply.
21. Anxiety or paranoia
22. Chest pain
23. Change in appetite
24. Dizziness
25. Euphoria or heightened sense of well-being
26. Fatigue or drowsiness
27. Headache
28. Increased blood pressure
29. Nausea or vomiting
30. Numbness or tingling
31. Palpitations
32. Shortness of breath
33. Sleeplessness
34. Weakness
35. Other (please specify)
36. [If Yes to 2] Why have you taken supplements for the purpose of improving your cognitive performance? Select all that apply.
37. To treat symptoms of a learning disorder or disability
38. To treat symptoms of another medical condition, such as attention deficit hyperactivity disorder (ADHD), shift work sleep disorder, narcolepsy, etc.
39. To improve concentration, memory, and/or alertness
40. To increase studying or working time
41. Another reason (please specify)

**Prescription medications**

1. Have you ever taken any of the following medications? Select yes or no for each.
2. Amphetamine (such as Adderall, Adzenys, Desoxyn, Dexedrine, Dyanavel, Evekeo, Mydayis, ProCentra, Vyvanse, or Zenzedi
3. Methylphenidate (such as Adhansia, Azstarys, Aptensio, Concerta, Contempla, Daytrana, Focalin, Jornay, Metadate, Methylin, QuilliChew, Quillivant, or Ritalin)
4. Modafinil (Provigil)
5. Cholinesterase inhibitor [such as Donepezil (Aricept), Rivastigmine (Exelon), or Galantamine (Razadyne)]
6. Glutamate regulator [Memantine (Namenda)]
7. Cholinesterase inhibitor + glutamate regulator [Donepezil and memantine (Namzaric)]

**Questions 9-11 will be asked for Amphetamine, Methylphenidate, and Modafinil selected above.**

1. At what stage of your training did you start taking [pipe in name of specific medication]?
2. Elementary school
3. Middle school
4. High school
5. College
6. Graduate school
7. Medical school
8. Residency
9. Other
10. How frequently are you currently taking [pipe in name of specific medication]?
11. No longer taking
12. Daily
13. At least once per week
14. At least once per month
15. At least once per year
16. Other (please specify)
17. Have you ever experienced side effects from taking [pipe in name of specific medication]?
18. Yes
19. No
20. [if yes to 11] What side effects have you experienced? Select all that apply.
21. Anxiety or paranoia
22. Chest pain
23. Change in appetite
24. Dizziness
25. Euphoria or heightened sense of well-being
26. Fatigue or drowsiness
27. Headache
28. Increased blood pressure
29. Nausea or vomiting
30. Numbness or tingling
31. Palpitations
32. Shortness of breath
33. Sleeplessness
34. Weakness
35. Other (please specify)
36. [If Yes to 8] Why have you taken medications for the purpose of improving your cognitive performance? Select all that apply.
37. To treat a learning disorder or disability
38. To treat another medical condition, such as attention deficit hyperactivity disorder (ADHD), shift work sleep disorder, narcolepsy, etc.
39. To improved concentration, memory, and/or alertness
40. To increased studying or working time
41. Another reason (please specify)

**Motivations**

1. To what extent do you agree or disagree with these statements? (response scale: strongly agree, somewhat agree, neither agree nor disagree, somewhat disagree, strongly disagree)
2. I feel pressure to perform well professionally and/or academically.
3. I feel afraid that I will be left behind professionally and/or academically.
4. To what extent do you agree or disagree with these statements? (response scale: strongly agree, somewhat agree, neither agree nor disagree, somewhat disagree, strongly disagree)
5. I feel pressure to take cognitive-enhancing supplements and/or medications because my colleagues take them.
6. I feel nervous about getting into trouble if I take cognitive-enhancing supplements and/or medications.
7. I feel nervous about the side effects of taking cognitive-enhancing supplements and/or medications.
8. I feel hesitant about the cost of buying cognitive-enhancing supplements and/or medications.

**Perceptions**

1. I could not have reached my current level of training without taking cognitive enhancing supplement(s) and/or medication(s). (response scale: strongly agree, somewhat agree, neither agree nor disagree, somewhat disagree, strongly disagree)
2. It is possible to achieve the level of academic or professional performance expected of me without taking cognitive enhancing supplement(s) and/or medication(s). (response scale: strongly agree, somewhat agree, neither agree nor disagree, somewhat disagree, strongly disagree)

**Demographic information**

1. Have you ever been diagnosed with the following medical conditions? Select yes or no for each.
2. Learning disorder or disability
3. Attention deficit hyperactivity disorder (ADHD)
4. Shift work sleep disorder
5. Narcolepsy
6. Sleep apnea with excessive daytime sleepiness
7. Neurodegenerative disease, such as Alzheimer’s disease or dementia
8. I identify my gender as
9. Female
10. Male
11. Trans-gender female
12. Trans-gender male
13. Gender queer
14. Non-binary
15. Other (please specify)
16. Prefer not to answer
17. Are you married?
18. Yes
19. No
20. Prefer not to answer
21. Do you have children?
22. Yes
23. No
24. Prefer not to answer
25. What is your primary specialty? Select all that apply.
26. Anatomic Pathology and Clinical Pathology
27. Anesthesiology
28. Child Neurology
29. Dermatology
30. Diagnostic Radiology
31. Emergency Medicine
32. Family Medicine
33. Internal Medicine
34. Internal Medicine-Pediatrics
35. Neurological Surgery
36. Neurology
37. Obstetrics and Gynecology
38. Occupational medicine
39. Ophthalmology
40. Orthopaedic Surgery
41. Otolaryngology
42. Pediatrics
43. Physical Medicine and Rehabilitation
44. Plastic Surgery
45. Psychiatry
46. Radiation Oncology
47. Surgery
48. Triple Board (Pediatrics, Psychiatry, Child and Adolescent Psychiatry)
49. Urology
50. Other (please specify)
51. Prefer not to answer
